# High-Resolution Multi-Channel Frequency Standard Comparator Using Digital Frequency Measurement

**DOI:** 10.3390/s21165626

**Published:** 2021-08-20

**Authors:** Bo Xiao, Ya Liu, Xiaohui Li, Zhifeng Deng, Yanrong Xue

**Affiliations:** 1 National Time Service Center, Chinese Academy of Sciences, East Shu Yuan Road, Xi’an 710600, China; xiaobo@ntsc.ac.cn (B.X.); xiaohui@ntsc.ac.cn (X.L.); dengzhifeng18@mails.ucas.ac.cn (Z.D.); xueyanrong@ntsc.ac.cn (Y.X.); 2 Key Laboratory of Time and Frequency Primary Standards, Chinese Academy of Sciences, Xi’an 710600, China; 3 School of Astronomy and Space Science, University of Chinese Academy of Sciences, Yu Quan Road, Beijing 100049, China

**Keywords:** multichannel, frequency standard comparison, digital frequency measurement

## Abstract

The rapid improvement accuracy of the atomic frequency standard puts forward higher requirements for the measurement resolution of the atomic frequency standard comparison system. To overcome the defect that the single zero-crossing point detection is sensitive to noise in the traditional dual mixer time difference measurement method, a digital frequency measurement method is proposed. This method combines sinusoidal beat technology, multi-channel synchronous acquisition technology, and digital frequency measurement technology, and uses differential compensation of system error to realize the precision measurement of atomic frequency standard. The frequency measurement accuracy is less than 2.5 × 10^−14^ and the noise floor is better than 6.5 × 10^−15^*/τ* = 1 s. The system has a high frequency measurement accuracy and a low noise floor, which can realize the precise measurement of a highly stable frequency source.

## 1. Introduction

Precise atomic frequency standard has provided time-frequency reference for modern communications, deep space exploration, satellite navigation [1,2]. However, due to the factors such as the aging of components and changes in the operating environment, the atomic frequency standard has a long-term drift during operation [3,4,5]. To achieve the state monitoring and performance evaluation of the atomic frequency standard, the development of the atomic frequency standard measurement and analysis system is of great significance. Besides, with the development of science and technology, the performance of atomic frequency standards is constantly improving [6]. For example, the accuracy of a new type of atomic frequency standard can reach 1 × 10^−15^, the uncertainty of the optical clock can even reach 1 × 10^−18^/*d*, and the stability of an ultra-stable crystal oscillator can reach 1 × 10^−14^/*τ* = 1 s [7,8,9], which puts forward higher requirements for the measurement resolution of atomic frequency standard measurement and analysis equipment.

High-precision frequency standard comparison methods usually use phase comparison technology. Among them, the dual mixer time difference (DMTD) method is widely used for atomic frequency standard comparison, and many precision frequency measurement equipments have been developed based on this method. For example, to provide time-frequency reference laboratories with real-time monitoring and performance testing of atomic clocks, TimeTech has developed a Phase-Comp [10]. The Phase-Comp uses two time interval counters to measure the time interval between zero-crossing points through coarse and fine measurements. To meet the needs of deep space exploration, NASA Jet Propulsion Experiment (JPL) has developed a multi-channel Frequency Standard Stability Analyzer (FSSA) [11,12]. When the beat signal triggers the zero-crossing event, the FSSA uses the event counter to mark the time stamp to obtain the phase difference between the zero-crossing points. In addition, there is a Software Defined Radio (SDR) frequency standard comparator, which is a fully digital DMTD [13]. There is the TSC-5125A phase noise tester from MICROSEMI of the United States and the VCH-323 phase comparison analyzer from VREMYA-CH of Russia [14,15], which use a fully digital dual mixer time difference method to perform analog-to-digital conversion on the input signal directly. Through digital signal processing, the phase difference between the signal under test and the reference signal is measured. The performance parameters of each frequency standard comparison equipment are shown in Table 1.

To overcome the defect that the single zero-crossing point detection is sensitive to noise in the traditional dual mixer time difference measurement method, this paper proposes a digital frequency measurement method. Different from fully digital DMTD in Software Defined Radio such as TSC-5125A or VCH-323, the method performs A/D sampling on the analog sinusoidal beat signal and uses a digital signal processing method to measure the frequency of the sinusoidal beat signal. This article proposes two frequency measurement methods: the cross-correlation decimal frequency measurement method and the three-point integer frequency measurement method. The paper [16] also mentioned the same method, but it uses the acquisition card of National Instrument (NI) to sample the sinusoidal beat signal. On the one hand, the data acquisition card requires initial calibration, and the calibration results may affect the accuracy of the signal measurement results. On the other hand, the data acquisition card is difficult to expand the channel. This paper presents a hardware implementation scheme of data acquisition and analysis based on ZYNQ-7020. The scheme has a low design cost and is easy to expand the channel. This scheme also has lower design costs and better frequency comparison performance than the data acquisition card.

To study the high-resolution atomic frequency standard comparison method, this paper first analyzes the basic principle of the DMTD method and factors affecting the measurement resolution. Then the frequency measurement algorithm is introduced in detail. Further, a hardware implementation is given. Simultaneously, an experimental test platform is created, the frequency measurement accuracy and noise floor of the measurement system based on the two methods are analyzed. Ultimately, we analyze the test results, and the discussion is given.

## 2. The Principle of DMTD Method

**Figure 1:**
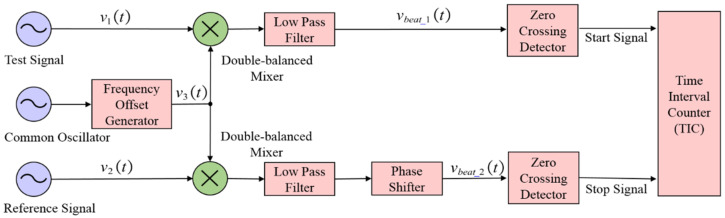
A schematic diagram of the DMTD method.

The DMTD method is a high-precision phase comparison technique that can be used to evaluate signal stability [17,18,19]. As can be seen from Figure 1, the test signal $v_{1}\left(t\right)$ with the nominal frequency $f_{0}$ and reference signal $v_{2}\left(t\right)$ with the same nominal frequency shown in the following formula is connected to the input port of the DMTD measurement system for time difference measurement.

(1) $$v_{i}\left(t\right)=A_{i}\left(t\right)\sin\left\{2\pi \left(f_{0}+\Delta f_{i}\left(t\right)\right)t+\theta_{i}\right\},i=1,2$$

where $A_{i}\left(t\right)$ is the amplitude of each signal. $\theta_{i}$ is the initial phase. $\Delta f_{i}\left(t\right)$ is a smooth real function, which reflects the phase noise of the signal.

In the DMTD method, a frequency offset generator (FOG) is used as shown in Figure 1. The FOG outputs the frequency offset signal $v_{3}\left(t\right)$ with the frequency $f_{c}$, which has a frequency offset $f_{beat}$ compared to $f_{0}$. The frequency offset signal $v_{3}\left(t\right)$ can be expressed by the formula as below:

(2) $$v_{3}\left(t\right)=A_{3}\left(t\right)\sin\left\{2\pi \left(f_{c}+\Delta f_{c}\left(t\right)\right)t+\theta_{3}\right\}$$

The test signal and reference signal are mixed with the frequency offset signal through a double-balanced mixer. The signals output from the two double-balanced mixers are processed by a low-pass filter to generate the sinusoidal beat signals $v_{beat\_i}\left(t\right)$.

(3) $$v_{beat\_i}=B_{i}\left(t\right)\sin\left\{2\pi \left(f_{beat}t+\left[\Delta f_{i}\left(t\right)-\Delta f_{c}\left(t\right)\right]t\right)\right\}$$

where $B_{i}\left(t\right)>0$ is the amplitude of each beat signal. $f_{beat}$ is a nominal frequency of the sinusoidal beat signal.

After two beat signals are detected at the zero-crossing detector, it is provided as the start signal and stop signal to the time interval counter (TIC) for time difference measurement.

Assuming that $t_{k},\;t_{k}^{\prime }$ respectively denotes the time when the beat signals $v_{beat\_1}\left(t\right)$ and $v_{beat\_2}\left(t\right)$ cross zero for the k-th time, then the following formulas are obtained:

(4) $$k=f_{beat}t_{k}+\left[\Delta f_{1}\left(t_{k}\right)-\Delta f_{c}\left(t_{k}\right)\right]t_{k}$$

(5) $$k=f_{beat}t_{k}^{\prime }+\left[\Delta f_{2}\left(t_{k}^{\prime }\right)-\Delta f_{c}\left(t_{k}^{\prime }\right)\right]t_{k}^{\prime }$$

Therefore, the time interval between two beating signals measured by TIC is $\Delta t_{k}=t_{k}-t_{k}^{\prime }$.

(6) $$\Delta t_{k}=\frac{\Delta f_{1}\left(t_{k}\right)t_{k}-\Delta f_{2}\left(t_{k}^{\prime }\right)t_{k}^{\prime }}{f_{beat}}+\frac{\Delta f_{c}\left(t_{k}^{\prime }\right)t_{k}^{\prime }-\Delta f_{c}\left(t_{k}\right)t_{k}}{f_{beat}}$$

The first term of the above formula denotes the effect of frequency fluctuation of the test signal and reference signal on the time interval between two beat signals. The second term shows that although the symmetric structure of DMTD can eliminate most of the noise from the frequency offset signal, it still contains the residual amount of the noise from the frequency offset signal [20,21]. To reduce the influence of noise as much as possible, a phase shifter is added to the reference signal channel to reduce the phase difference between the reference signal and the test signal. Then, the $\Delta t_{k}$ can be approximately expressed as:

(7) $$\begin{array}{c} \Delta t_{k}\approx \frac{\Delta f_{1}\left(t_{k}\right)t_{k}-\Delta f_{2}\left(t_{k}^{\prime }\right)t_{k}^{\prime }}{f_{beat}}\\ \approx \frac{f_{0}}{f_{beat}}\cdot \frac{\Delta f_{1}\left(t_{k}\right)t_{k}-\Delta f_{2}\left(t_{k}^{\prime }\right)t_{k}^{\prime }}{f_{0}} \end{array}$$

The second term of the multiplier factor in Equation (7) denotes the time difference ΔT between the test signal and the reference signal. It also shows that the measurement resolution ΔT is enhanced by a factor of $f_{0}/f_{beat}$.

In addition to noise from the frequency offset signal, there is also random noise generated by each device in the DMTD system [22]. Since the zero-crossing point detection is sensitive to noise, it is easy to lead to misjudgment of the zero-crossing point and cause measurement errors, which limit the improvement of the measurement resolution of DMTD method.

## 3. Method of Digital Frequency Measurement

**Figure 2:**
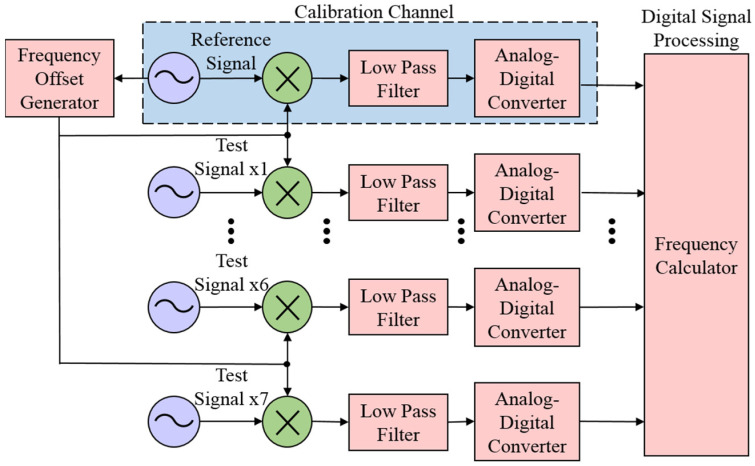
A schematic diagram of the multi-channel digital frequency measurement system.

To reduce the influence of noise superimposed on a single zero-crossing point, a multi-channel digital frequency measurement method is proposed [16]. Compared with the DMTD method, the proposed method performs analog-to-digital conversion on the sinusoidal beat signal output by the low-pass filter instead of zero-crossing detection. Multi-point sampling (including zero-crossing point) of the beat signal can reduce the influence of noise on the measurement result more than single-point zero-crossing detection [23]. In addition, the design of multi-channel ensures that the signals of each channel can be measured synchronously, to calibrate the error of the measurement results. On the other hand, it can also meet the needs of simultaneous measurement of multiple test signals. The multi-channel digital frequency measurement system is displayed in Figure 2.

The multi-channel digital frequency measurement system consists of one calibration channel and seven measuring channels, and the number of measuring channels can be arbitrarily extended. The circuit structure of the calibration channel is consistent with each measurement channel. In the process of data acquisition, the Analog-Digital Converter of each channel should use a common sampling clock to ensure that the frequency measurement results are simultaneous, it also ensures the strong correlation of the noise of each channel. 

Different from the measurement channel, the input signal of the calibration channel is also a reference input of the frequency offset generator. Due to the consistency of circuit structure and strong correlation of noise, the measurement error of the beat signal frequency of the calibration channel can represent the systematic error. Then the frequency measurement results of each measurement channel can be subtracted from that of the calibration channel to eliminate the influence of additional measurement noise.

The calculation of frequency adopts digital signal processing methods, including cross-correlation decimal frequency measurement and three-point integer frequency measurement.

The discrete sinusoidal beat signal of each channel is shown in the following formula:

(8) $$v_{i}\left(n\right)=V_{i}\sin\left(2\pi \frac{f_{beat}+\Delta f_{i}}{f_{s}}n+\varphi_{i}\right)+N_{i}\left(n\right)$$

where $V_{i}$ is the amplitude of the beat signal of channel *i*. $f_{beat}$ is the nominal frequency of the digital beat signal. $\Delta f_{i}$ is the frequency deviation of the input signal of channel *i*. The frequency deviation includes the integer component $\Delta f_{int}$ and fractional component $\Delta f_{dec}$, i.e., $\Delta f_{i}=\Delta f_{int\_i}+\Delta f_{dec\_i}$. $f_{s}$ is the sampling clock rate. $\varphi_{i}$ is the initial phase of the beat signal of channel i. n is the discrete time. $\varphi_{i}$ is the total noise of channel *i*.

### 3.1. Cross-Correlation Decimal Frequency Measurement

To calculate the decimal frequency deviation of the beat signal, the cross-correlation frequency measurement method extracts two groups of continuous sampling data with the length of $f_{s}$ from each channel for cross-correlation operation. It takes two seconds of sampled data to calculate $\Delta f_{dec\_i}$, therefore the calculated $\Delta f_{dec\_i}$ is the average decimal frequency deviation within the time interval of 2 s. The signals at the *j* and *j* + 1 second of channel *i* are expressed as follows:

(9) $$v_{i,j}\left(n\right)=V_{i,j}\sin\left(\omega_{i,j}n+\varphi_{i,j}\right)+N_{i,j}\left(n\right)$$

(10) $$v_{i,j+1}\left(n\right)=V_{i,j+1}\sin\left(\omega_{i,j}n+\varphi_{i,j+1}\right)+N_{i,j+1}\left(n\right)$$

where, $\omega_{i,j}=2\pi \frac{f_{beat}+\Delta f_{i,j}}{f_{s}}$, $\Delta f_{i,j}$ denotes the average frequency deviation between the *j* and *j*+1 second of channel *i*. $V_{i,j}$ and $V_{i,j+1}$ are the amplitude of the beat signal.

The cross-correlation result of $v_{i,j}\left(n\right)$ and $v_{i,j+1}\left(n\right)$ can be expressed by:

(11) $$\begin{array}{c} R_{v_{i,j},v_{i,j+1}}\left(\tau \right)=\frac{1}{f_{s}}\sum_{n=0}^{f_{s}-1}v_{i,j}\left(n\right)v_{i,j+1}\left(n+\tau \right)\\ =\frac{1}{2}V_{i,j}V_{i,j+1}\cos\left(2\pi \omega_{i,j}\cdot \tau +\Phi_{i,j}\right)\\ +R_{v_{i,j},N_{i,j+1}}+R_{v_{i,j+1},N_{i,j}}+R_{N_{i,j},N_{i,j+1}} \end{array}$$

where τ is the delay time between $v_{i,j}\left(n\right)$ and $v_{i,j+1}\left(n\right)$. $\Phi_{i,j}$ is the initial phase difference between $v_{i,j}\left(n\right)$ and $v_{i,j+1}\left(n\right)$ in the range of [−π,π], and $\Phi_{i,j}=\varphi_{i,j+1}-\varphi_{i,j}$. $R_{v_{i,j},N_{i,j+1}}$ and $R_{v_{i,j+1},N_{i,j}}$ denote the cross-correlation value between digital sinusoidal beat signal and noise. $R_{N_{i,j},N_{i,j+1}}$ denotes the cross-correlation value between noises. Statistically, the signal is not correlated with noise, i.e., $R_{v_{i,j},N_{i,j+1}}=0$, $R_{v_{i,j+1},N_{i,j}}=0$. There is also no correlation between the noises or the correlation is very small enough to be ignored, i.e., $R_{N_{i,j},N_{i,j+1}}\approx 0$.

When τ=0 Equation (11) can be expressed as:

(12) $$R_{v_{i,j},v_{i,j+1}}\left(0\right)=\frac{1}{2}V_{i,j}V_{i,j+1}\cos\left(\Phi_{i,j}\right)$$

If $v_{i,j}\left(n\right)$ and $v_{i,j+1}\left(n\right)$ performs cross-correlation operation with themselves respectively, $V_{i,j}$ and $V_{i,j+1}$ can be expressed by:

(13) $$V_{i,j}=\sqrt{2R_{v_{i,j},v_{i,j}}\left(0\right)}$$

(14) $$V_{i,j+1}=\sqrt{2R_{v_{i,j+1},v_{i,j+1}}\left(0\right)}$$

According to the Formula (12)–(14), the $\Phi_{i,j}$ can be expressed as follows:

(15) $$\Phi_{i,j}=\pm \arccos\left(\frac{R_{v_{i,j},v_{i,j+1}}\left(0\right)}{\sqrt{R_{v_{i,j},v_{i,j}}\left(0\right)\cdot R_{v_{i,j+1},v_{i,j+1}}\left(0\right)}}\right)$$

Since $v_{i,j}\left(n\right)$ and $v_{i,j+1}\left(n\right)$ are two groups of continuous sampling data, the initial phase $v_{i,j+1}\left(n\right)$ is equal to the phase of $v_{i,j}\left(n\right)$ at $n=f_{s}$. As shown in the follows:

(16) $$\varphi_{i,j+1}=\varphi_{i,j}+2\pi \left(\frac{f_{beat}+\Delta f_{i,j}}{f_{s}}\cdot f_{s}\right)$$

$f_{beat}$ is an integer, and $\Delta f_{i,j}=\Delta f_{int\_i,j}+\Delta f_{dec\_i,j}$, the initial phase difference $\Phi_{i,j}$ can be expressed as:

(17) $$\Phi_{i,j}=\varphi_{i,j+1}-\varphi_{i,j}=2\pi \cdot \Delta f_{dec\_i,j}$$

Combining Formulas (15) and (17), $\Delta f_{dec\_i,j}$ can be expressed as:

(18) $$\left|\Delta f_{dec\_i,j}\right|=\frac{1}{2\pi }\arccos\left(\frac{R_{v_{i,j},v_{i,j+1}}\left(0\right)}{\sqrt{R_{v_{i,j},v_{i,j}}\left(0\right)\cdot R_{v_{i,j+1},v_{i,j+1}}\left(0\right)}}\right)$$

The fractional part of frequency deviation can be measured by the cross-correlation decimal frequency measurement method. There is only multiplication and addition in the cross-correlation algorithm, so the method is simple and easy to implement with Field Programmable Gate Array (FPGA), which not only meets the requirements of algorithm implementation, but it also supports multi-channel parallel measurement. The numerical range of $\Delta f_{dec\_i,j}$ is [−0.5,0.5]. As for the determination of the sign $\Delta f_{dec\_i,j}$, a detailed description will be given later.

### 3.2. Three-Point Integer Frequency Measurement

Three-point integer frequency measurement can measure the integer part of beat signal frequency, including nominal frequency $f_{beat}$ and integer frequency deviation $\Delta f_{int}$. According to the trigonometric identities, the beating signal of channel *i* at *j* seconds expressed by Formula (9) has the following relationship:

(19) $$v_{i,j}\left(n\right)=2\cos\left(\omega_{i,j}\right)\cdot v_{i,j}\left(n-1\right)-v_{i,j}\left(n-2\right)$$

where $\omega_{i,j}=2\pi \frac{f_{beat}+\Delta f_{i,j}}{f_{s}}$, $\Delta f_{i,j}=\Delta f_{int\_i}+\Delta f_{dec\_i}$ denotes the average frequency deviation at *j* second.

Therefore, the frequency of the beat signal can be expressed as follows:

(20) $$f_{beat}+\Delta f_{i,j}=\frac{f_{s}}{2\pi }\arccos\left(\frac{v_{i,j}\left(n\right)+v_{i,j}\left(n-2\right)}{2\cdot v_{i,j}\left(n-1\right)}\right)$$

Generally, the frequency value of the digital beat signal can be calculated as long as three consecutive sampling points are obtained. However, in the process of sampling and digitizing, there will be cases where the sampling data is zero. The above methods need to be improved.

If the sampling data $v_{i,j}\left(n-1\right)$ and $v_{i,j}\left(n-2\right)$ are used to estimate the data $v_{i,j}\left(n\right)$ according to Formula (2), the estimated value ${\hat{v}}_{i,j}\left(n\right)$ can be expressed as:

(21) $${\hat{v}}_{i,j}\left(n\right)=c\cdot v_{i,j}\left(n-1\right)-v_{i,j}\left(n-2\right)$$

where $c=2\cos\left(2\pi \frac{{\hat{f}}_{beat}+\Delta {\hat{f}}_{i,j}}{f_{s}}\right)$, ${\hat{f}}_{beat}+\Delta {\hat{f}}_{i,j}$ is the estimated frequency of the beat signal. Then the estimation error can be expressed as:

(22) $$\begin{array}{c} e_{i,j}\left(n\right)=v_{i,j}\left(n\right)-{\hat{v}}_{i,j}\left(n\right)\\ =v_{i,j}\left(n\right)-c\cdot v_{i,j}\left(n-1\right)-v_{i,j}\left(n-2\right) \end{array}$$

When the estimation error $e_{i,j}\left(n\right)$ is minimum, it means that ${\hat{f}}_{beat}+\Delta {\hat{f}}_{i,j}$ is the optimal estimated frequency of the beat signal. Then the following equation holds:

(23) $$\frac{\partial E\left({\left|e_{i,j}\left(n\right)\right|}^{2}\right)}{\partial c}=0$$

According to Formula (23), the estimated frequency can be calculated as follows:

(24) $$c=\frac{\sum_{3}^{N}\left[v_{i,j}\left(n\right)+v_{i,j}\left(n-2\right)\right]v_{i,j}\left(n-1\right)}{\sum_{3}^{N}v_{i,j}\left(n-1\right)v_{i,j}\left(n-1\right)}$$

(25) $${\hat{f}}_{beat}+\Delta {\hat{f}}_{i,j}=\frac{f_{s}}{2\pi }\arccos\left(\frac{c}{2}\right)$$

where *N* denotes the number of sample data participating in the operation. The integer frequency of the beat signal can be expressed as follows:

(26) $$f_{int\_i,j}=INT\left[{\hat{f}}_{beat}+\Delta {\hat{f}}_{i,j}\right]$$

where INT[∗] is the rounding function.

The integral part of frequency deviation can be measured by the three-point integer frequency measurement method. The multiplication and addition operation in this method is easy to implement with FPGA. The numerical range of $f_{int\_i,j}$ is $\left[0,f_{s}/2\right]$. Since the frequency measurement resolution is related to $f_{s}$. Care should be taken to select the appropriate $f_{s}$ to ensure the accuracy of integer measurement.

### 3.3. Frequency Measurement of the Digital Beat Signal

In practice, the digital beat signal has a positive or negative decimal frequency deviation, but the cross-correlation decimal frequency measurement can only calculate the absolute value of decimal frequency deviation. The sign of the value of $\Delta f_{dec\_i,j}$ can be judged with the help of a three-point integer frequency measurement result, and the frequency of the digital beat signal is finally expressed below as:

case (i): if $INT\left[{\hat{f}}_{beat}+\Delta {\hat{f}}_{i,j}\right]<INT\left[{\hat{f}}_{beat}+\Delta {\hat{f}}_{i,j}+0.5\right]$,
  then $f_{beat\_i,j}=f_{int\_i,j}-\left|\Delta f_{dec\_i,j}\right|+1$.
case (ii): if $INT\left[{\hat{f}}_{beat}+\Delta {\hat{f}}_{i,j}\right]=INT\left[{\hat{f}}_{beat}+\Delta {\hat{f}}_{i,j}+0.5\right]$,
  then $f_{beat\_i,j}=f_{int\_i,j}+\left|\Delta f_{dec\_i,j}\right|$.

According to the measured frequency value of the digital beat signal of each channel, the frequency deviation of the sinusoidal beat signal between each measurement channel and reference channel can be obtained. The result of dividing the frequency deviation of the sinusoidal beat signal by the factor $\frac{f_{0}}{f_{beat}}$ is the relative frequency deviation between the measured signal and the reference signal.

## 4. Hardware Implementation

A multi-channel digital frequency measurement method is proposed in Section 3. The specific hardware design of this method will be introduced in this section. We adopt a modular design to divide the hardware circuit into three parts: frequency offset generator, sinusoidal beat device, and multi-channel data acquisition and analysis module. A detailed description of each module is given below.

### 4.1. Frequency Offset Generator Module

**Figure 3:**
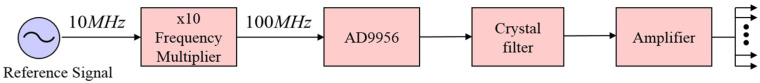
A block diagram of the frequency offset generator.

As shown in Figure 3, the frequency offset generation module uses direct digital synthesis technology to generate frequency offset signals through AD9956. The reference signal with the frequency of 10 MHz passes through the ×10 frequency multiplier, and the 100 MHz sinusoidal signal generated by the frequency multiplier provides the system clock to AD9956. Then the AD9956 generates a frequency offset signal with a frequency $10MHz-f_{beat}$, Usually the value of $f_{beat}$ is 1 Hz, 10 Hz, or 100 Hz. The crystal filter is a bandpass filter with a center frequency of 10 MHz and a passband range of 1 kHz. The in-band fluctuation of the filter is less than 1 dB. The amplifier uses the LMH6702 operational amplifier circuit to amplify the power of the frequency offset signal and divides one signal into 8 channels for output.

### 4.2. Sinusoidal Beat Device Module

**Figure 4:**
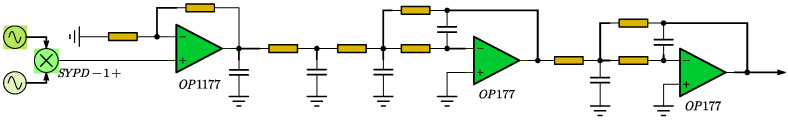
The circuit structure of the sinusoidal beat device module.

In the sinusoidal beat device, an SYPD-1+ phase detector with high sensitivity to phase fluctuation is selected as the double-balanced mixer, The measured signal is mixed with the frequency offset signal by SYPD-1+, and the primary amplification circuit composed of OP1177 amplifies the mixed signal. The bipolar multiple feedback active filter circuit composed of OP117 filters the mixed signal to select the low-frequency sinusoidal beat signal. The circuit structure of the sinusoidal beat device module is shown in Figure 4.

### 4.3. Multi-Channel Data Acquisition and Analysis Module

**Figure 5:**
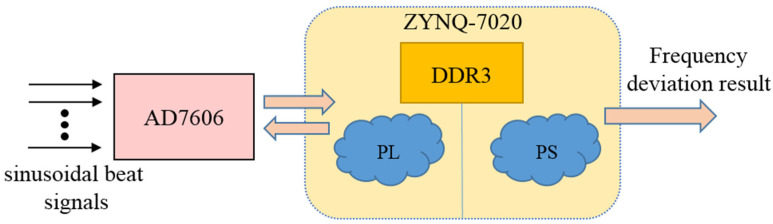
A block diagram of multi-channel data acquisition and analysis module.

As shown in Figure 5, the analog-to-digital data acquisition system (DAS) is realized by AD7606, which supports 8 synchronous sampling inputs. The resolution of AD7606 is 16 bits, and the sampling rate of all channels is 200kSPS. The processor adopts Xilinx ZYNQ-7020 System-on-Chip (SoC) with a dual-core ARM Cortex-A9 processor and FPGA, which is divided into programmable logic (PL) and processor system (PS). The PL of ZYNQ-7020 drives AD7606 to realize data acquisition, and the collected data is transmitted to DDR3 SDRAM through direct memory access (DMA). In addition, the PL also performs multiplication and addition operations in the frequency measurement algorithm on the collected data. The PS of ZYNQ-7020. The PS of ZYNQ-7020 performs division and inverse cosine operation on the multiplication and addition result, and finally calculates the frequency deviation of sinusoidal beat signal between each measurement channel and reference channel. 

## 5. Experimental Testing 

**Figure 6:**
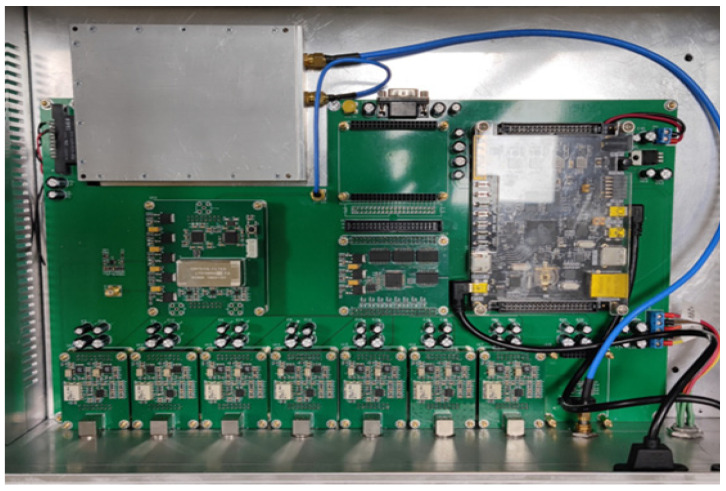
The structure of the multi-channel digital frequency standard comparison.

To evaluate the performance of the multi-channel digital frequency measurement method proposed in this article, a multi-channel digital frequency standard comparison prototype has been developed. The prototype consists of one calibration channel and seven measurement channels. The nominal frequency of the sinusoidal beat signal is 1 Hz, and the sampling clock of ADC is 200 kHz. Figure 6 presents the structure of the multi-channel digital frequency standard comparison prototype.

**Figure 7:**
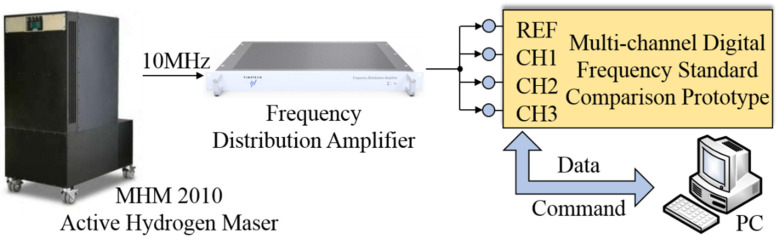
A diagram of the experimental test platform.

We have also established an experimental test platform to evaluate the frequency measurement accuracy and noise floor of the prototype. Figure 7 presents the experimental test platform diagram. A sinusoidal signal generated by an active hydrogen maser with a nominal frequency of 10 MHz is distributed to four channels by the frequency distribution amplifier. Then four sinusoidal signals are connected to the input port of one calibration channel and three measurement channels respectively. A Personal Computer (PC) is used to send commands to the prototype and receive the data of frequency calculation results.

### 5.1. Measurement Accuracy

**Figure 8:**
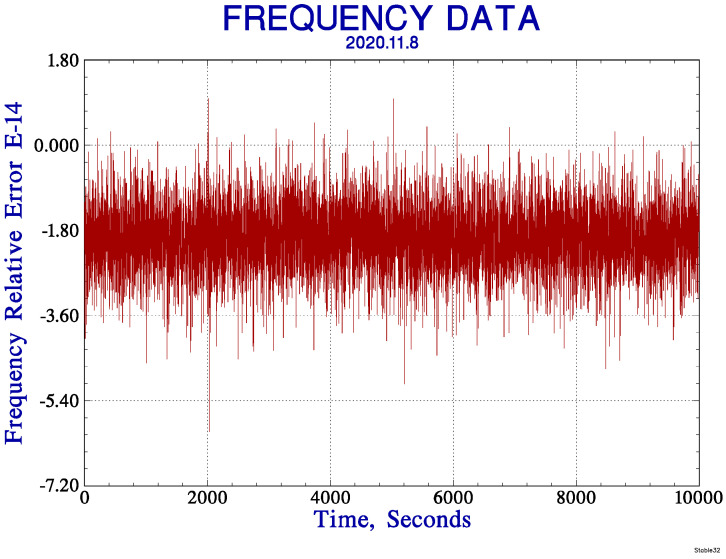
The frequency relative error during the continuous measurement of 10,000 s.

The accuracy can be used to evaluate the measurement ability of the multi-channel digital frequency standard comparison prototype, which can be characterized by relative error. The relative error can be measured by the quotient of the measurement error and the nominal value. Table 2 lists the frequency measurement average and relative error of CH1 in different time intervals. Figure 8 shows the relative error of the frequency during the continuous measurement of 10,000 s by Stable32 software. Table 2 and Figure 8 show that the relative error of frequency measurement of the prototype is less than 2.5 × 10^−14^, the error is very small, and the measurement accuracy is high, which can meet the needs of high-precision measurement.

### 5.2. Noise Floor

**Figure 9:**
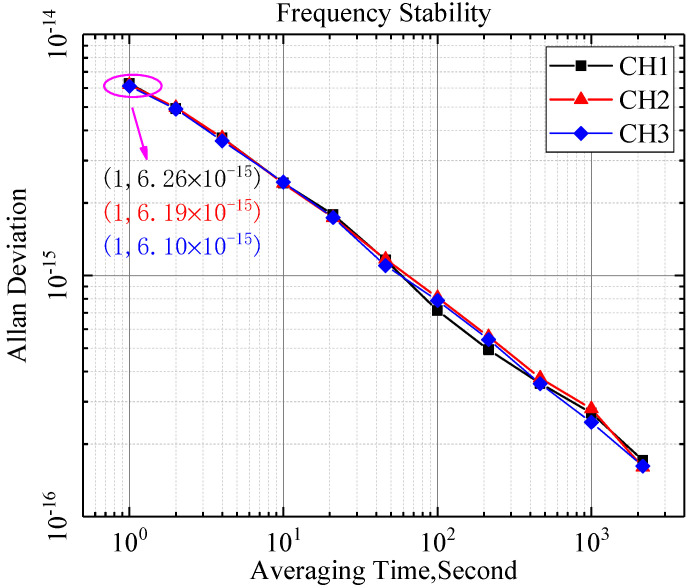
The frequency stability of three measurement channels with systematic error elimination.

**Figure 10:**
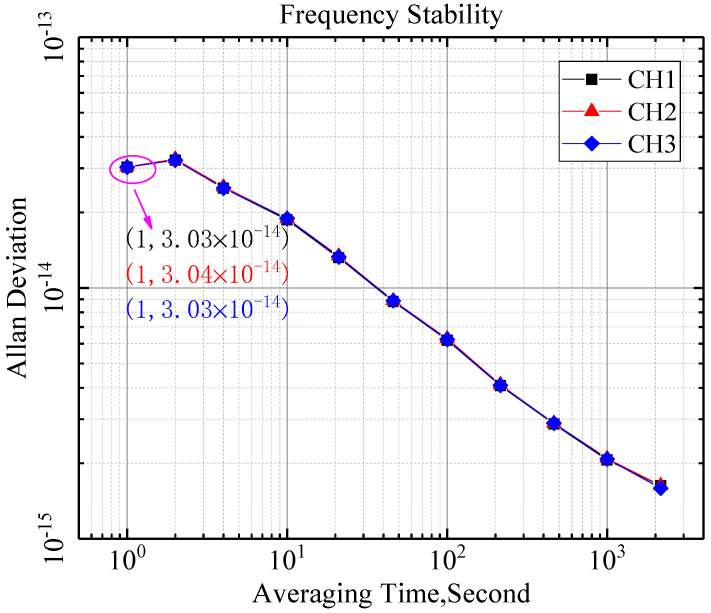
The frequency stability of three measurement channels without systematic error elimination.

Noise Floor refers to the additional noise of the measuring equipment itself except the useful signal, which is usually characterized by Allan variance at 1 s in the field of time and frequency. Figure 9 shows the frequency stability of three measurement channels with systematic error elimination. For an input signal with the frequency 10 MHz, the noise floor test results of the three measurement channels of the prototype are 6.26 × 10^−15^/*τ* = 1 s, 6.19 × 10^−15^/*τ* = 1 s, and 6.10 × 10^−15^/*τ* = 1 s. Compared with Figure 10, the measurement results of the measurement channel are subtracted from the calibration channel, which eliminates the influence of noise from the device and improves the performance of frequency standard comparison. The noise floor of the prototype is low enough to be used to evaluate the ultra-stable frequency source with frequency stability of 1 × 10^−14^/*τ* = 1 s. Simultaneously, the Allan variance curves of the three channels are approximately coincident, which also shows that there is a high consistency between the channels.

## 6. Conclusions and Discussion

The fast-developing high-precision clock sources are an important part of the time-frequency system. The Atomic clock usually provides time-frequency references for timekeeping systems in the form of clock groups, and it is of great practical significance to evaluate their frequency stability. In this study, a digital frequency measurement method is proposed. The performance of this method has been rigorously assessed.

In the prototype test, the relative error can reach 10^−14^ Hz. This is due to the multi-channel synchronous sampling of the prototype, which ensures that the system error of each channel has a strong correlation. Therefore, the system error of the calibration channel, whose structure is consistent with the measurement channel, can be used to compensate for the measurement results from the measurement channel.

The noise floor of the prototype is equivalent to that of existing products, and it can also support multi-channel simultaneous testing. Since the prototype only supports the signal with a nominal frequency of 10 MHz, it is necessary to increase the frequency measurement bandwidth of the system to expand the application range in subsequent work.

## Figures and Tables

**Table 1:** The performance parameters of each frequency standard comparison equipment.

Equipment	Number of Channels	Noise Floor (ADEV *τ* = 1 s)
Phase-Comp	6~16	2.5 × 10^−14^(@10 MHz)
FSSA	8	2.0 × 10^−15^(@100 MHz)
TSC-5125A	2	3.0 × 10^−15^(@10 MHz)
VCH-323	3	7.0 × 10^−15^

**Table 2:** The requency measurement average and relative error.

Time (s)	Frequency (Hz)	Relative Error
1	9,999,999.999999756	−2.44 × 10^−14^
3	9,999,999.999999785	−2.15 × 10^−14^
10	9,999,999.999999798	−2.02 × 10^−14^
30	9,999,999.999999803	−1.97 × 10^−14^
100	9,999,999.999999809	−1.91 × 10^−14^
300	9,999,999.999999803	−1.97 × 10^−14^
1000	9,999,999.999999806	−1.94 × 10^−14^
3000	9,999,999.999999805	−1.95 × 10^−14^
10,000	9,999,999.999999804	−1.96 × 10^−14^

## Data Availability

Not applicable.
